# Prevalence of Unrecognized Cognitive Impairment in Federally Qualified Health Centers

**DOI:** 10.1001/jamanetworkopen.2024.40411

**Published:** 2024-10-22

**Authors:** Ambar Kulshreshtha, Erik S. Parker, Nicole R. Fowler, Diana Summanwar, Zina Ben Miled, Arthur H. Owora, James E. Galvin, Malaz A. Boustani

**Affiliations:** 1 Department of Family and Preventive Medicine, Emory University, Atlanta, Georgia; 2 Department of Epidemiology and Biostatistics, Indiana University School of Public Health, Bloomington; 3 Department of Medicine, Indiana University School of Medicine, Indianapolis; 4 Indiana University Center for Aging Research, Indianapolis; 5 Regenstrief Institute, Inc, Indianapolis, Indiana; 6 Center for Health Innovation and Implementation Science, Indiana University School of Medicine, Indianapolis; 7 Department of Family Medicine, Indiana University School of Medicine, Indianapolis; 8 Electrical and Computer Engineering, Indiana University-Purdue University, Indianapolis; 9 Department of Pediatrics, Indiana University School of Medicine, Indianapolis; 10 Comprehensive Center for Brain Health, Department of Neurology, University of Miami Miller School of Medicine, Boca Raton, Florida

## Abstract

**Importance:**

There is a need for early and equitable detection of cognitive impairment among older adults.

**Objective:**

To examine the prevalence of unrecognized cognitive impairment among older adults receiving primary care from federally qualified health centers (FQHCs).

**Design, Setting, and Participants:**

This cross-sectional study was conducted at 5 FQHCs providing primary care in Indianapolis, Indiana, between 2021 and 2023. Participants were adults aged 65 years and older, without a diagnosis of mild cognitive impairment (MCI), dementia, or severe mental illness. Data analysis was performed from September 2023 to April 2024.

**Main Outcomes and Measures:**

The primary outcome was a diagnosis of dementia or MCI, as determined by an interdisciplinary clinical team using data from structured patient and study partner interviews, medical record reviews, and a detailed cognitive assessment, including neuropsychological testing. Differences between participants determined to have normal cognition, MCI, and dementia were assessed statistically using analysis of variance for continuous variables, χ^2^ or Fisher exact tests for categorical variables, or Fisher exact test alone when expected cell counts were 5 or less.

**Results:**

A total of 844 eligible individuals were consecutively approached, 294 consented to participate, and 204 completed the study (mean [SD] age, 70.0 [5.1] years; 127 women [62.3%]). One hundred eight participants (52.9%) were African American, 5 (2.5%) were Hispanic, 199 (97.5%) were not Hispanic, and 90 (44.1%) were White. The mean (SD) duration of education was 13.1 (2.6) years, and the mean (SD) Area Deprivation Index score was 78.3 (19.9), indicating a high level of neighborhood disadvantage. In total, 127 patients (62.3%) met the diagnostic criteria for MCI, 25 (12.3%) had dementia, and 52 (25.5%) had no cognitive impairment. Compared with non-Hispanic White individuals and after adjusting for age, sex, and education level, African American individuals were more than twice as likely to have MCI or dementia (odds ratio, 2.73; 95% CI, 1.38-5.53; *P* = .02).

**Conclusions and Relevance:**

This cross-sectional study found that unrecognized cognitive impairment is ubiquitous among older adults from underrepresented, minoritized racial and ethnic groups and those who are socially vulnerable receiving primary care from FQHCs. To overcome the disparity in early detection of cognitive impairment, timely, equitable, scalable, and sustainable detection approaches need to be developed.

## Key Points

**Question:**

What is the prevalence of unrecognized cognitive impairment among patients at federally qualified health centers?

**Findings:**

In this cross-sectional study of 204 adults aged 65 years or older with no prior diagnosis of cognitive impairment receiving primary care at federally qualified health centers, 62.3% had mild cognitive impairment, 12.3% had dementia, and only 25.5% had no cognitive impairment. African American individuals had more than double the odds of White individuals of having a diagnosis of mild cognitive impairment or dementia.

**Meaning:**

These findings suggest that unrecognized cognitive impairment is ubiquitous among individuals from underrepresented minoritized groups and socially vulnerable older adults receiving primary care from federally qualified health centers; there is a need to develop timely, equitable, scalable, and sustainable approaches for early detection of cognitive impairment.

## Teaser

This cross-sectional study examines the prevalence of unrecognized cognitive impairment among adults aged 65 years and older receiving primary care from federally qualified health centers in Indianapolis, Indiana.

## Introduction

There are racial, ethnic, and socioeconomic disparities in detecting Alzheimer disease and related dementia (ADRD), leading to a major impact on society.^1,2,3^ For example, compared with non-Hispanic White individuals, both African American and Hispanic individuals are at higher risk of developing ADRD and receive the diagnosis at a more advanced stage.^4^ There are also differences in ADRD associated with individual-level demographic characteristics, such as income and education.^5^ With the recent breakthroughs in ADRD treatment, such as the Food and Drug Administration’s approval of a new class of disease-modifying treatments for Alzheimer disease and the Centers for Medicare & Medicaid Services’ approval of the Guiding an Improved Dementia Experience model as an alternative payment model for comprehensive dementia care model, equitable early detection of ADRD is critical.^6,7,8^

Primary care settings are essential for equitable detection, treatment, and care of ADRD, yet substantial gaps persist owing to resource, time, and training constraints.^9,10,11,12^ Early detection of ADRD in primary care is particularly crucial for socially vulnerable populations and people living in underserved geographic regions who may have limited access to specialists.^13^ Federally qualified health centers (FQHCs) are nonprofit health centers that predominantly serve low-income and medically underserved communities. Three-quarters of their patients have family incomes less than 200% of the federal poverty level.^14^ FQHCs deliver comprehensive primary care services to more than 30 million individuals, including a growing number of older patients. The extent to which mild cognitive impairment (MCI) and dementia are unrecognized in these unique settings has not been examined. Using a comprehensive cognitive assessment,^15^ this study measured the prevalence of unrecognized and undiagnosed MCI and dementia among patients at FQHCs and examined its association with race. We hypothesized that the rate of unrecognized cognitive impairment would be higher among African American individuals than non-Hispanic White individuals.

## Methods

### Study Design and Eligibility

We conducted a cross-sectional observational study that consecutively enrolled and obtained consent from participants from 5 FQHC clinics affiliated with Eskenazi Health (Indianapolis, Indiana) from 2021 to 2023. Individuals were considered eligible for this study if they were patients at 1 of the 5 FQHCs involved in this study, aged 65 years or older, had at least 1 visit to an Eskenazi Health primary care practitioner in the preceding year, had 3 years of available electronic health records (EHRs), and were capable of giving verbal or written informed consent in English. The study excluded individuals with a documented diagnosis of dementia or MCI, as determined by the *International Statistical Classification of Diseases and Related Health Problems, Tenth Revision* code in their EHR, any history of prescription for a cholinesterase inhibitor or memantine, serious mental illness such as schizophrenia, or permanent residence in a nursing facility. Participants’ race and ethnicity (African American, Hispanic, or White) were based on the information in their EHRs and are included in this study to understand racial differences and ensure that our findings are generalizable to more communities.

Area Deprivation Index (ADI) scores are indexed to the national level and are the 2021 versions as obtained from the Neighborhood Atlas at the University of Wisconsin, Madison.^16^ The ADI scores range from 1 to 100, with higher scores denoting higher levels of neighborhood disadvantage.

 This study was approved by the institutional review board of Indiana University. We followed the Strengthening the Reporting of Observational Studies in Epidemiology (STROBE) reporting guidelines for cross-sectional studies.

### Cognitive Assessment

Once they provided consent, all participants underwent a comprehensive assessment that included the completion of structured patient and study partner interviews, medical record reviews, detailed neurological examination, and neuropsychological testing using the Uniform Data Set 3.0 protocol.^17^ The Montreal Cognitive Assessment (score range, 0-30, with higher scores denoting better cognitive performance) was used to assess global cognitive performance. The Clinical Dementia Rating (score range, 0-3, with higher scores denoting more cognitive impairment) was used to assess the presence or absence of cognitive impairment and, if present, grade its severity. The Neuropsychiatric Inventory Questionnaire (score range, 0-36, with higher scores denoting worse behavioral symptoms) was used to assess behavioral and psychological symptoms of dementia and informant distress. The Geriatric Depression Scale (score range, 0-15, with higher scores denoting more-severe depressive symptoms) was used to assess mood. The Functional Activity Questionnaire (score range, 0-30, with higher scores denoting greater functional impairment) was used to assess activities of daily living. An interdisciplinary clinical team of a neurologist (J.E.G.), a geriatrician (M.A.B.), and a neuropsychologist (not a coauthor of this article) reviewed all assessment and neuropsychological data and made a consensus-based diagnosis of normal cognition, MCI, or dementia, in accordance with the Uniform Data Set 3.0 protocol.^17^ The comprehensive clinical and neurocognitive assessment for the consensus diagnosis included adjustments for each patient’s education, sensory disorders, primary language, and comorbid conditions.

### Statistical Analysis

Data analysis was performed from September 2023 to April 2024. Means, medians, SDs, and ranges were calculated for all continuous descriptive variables. Categorical descriptive variables were represented as counts and percentages. Differences between participants determined to have normal cognition, MCI, and dementia were assessed statistically using analysis of variance for continuous variables, χ^2^ or Fisher exact tests for categorical variables, or the Fisher exact test alone when expected cell counts were 5 or less.

Multivariable logistic regression was conducted to examine whether the association between race and cognition differed by age, sex, or level of education by examining the significance of pairwise terms (eg, age by race, or sex by race). All reported tests were 2-sided, and *P* < .05 was considered significant. Data were analyzed using R statistical software version 4.3.0 (R Project for Statistical Computing).

## Results

We approached 844 eligible individuals for the study. Among those, 294 (34.8%) consented to participate and 204 completed the study (69.4%; mean [SD] age, 70.0 [5.1] years; 127 women [62.3%]). There were no demographic differences between the participants who refused the study and those who consented. Table 1 presents the demographic characteristics of the 204 patients who completed the study by cognitive status. One hundred eight participants (52.9%) were African American, 5 (2.5%) were Hispanic, 199 (97.5%) were not Hispanic, and 90 (44.1%) were White. The mean (SD) duration of education was 13.1 (2.6) years, and the mean (SD) ADI was 78.3 (19.9), indicating a high level of neighborhood disadvantage. Patients with dementia had significantly fewer years of education and had a higher ADI score compared with patients with no cognitive impairment. The consensus diagnoses for the cohort were 52 patients (25.5%) with no cognitive impairment, 127 patients (62.3%) with MCI, and 25 patients (12.3%) with dementia. No patients had moderate or severe dementia. Cardiovascular risk factors and mental health conditions such as depression were highly prevalent in the full sample. Table 2 describes the cognitive, functional, behavioral, and psychological performance of the total sample.

**Table 1.** Descriptive Summary of Demographic and Clinical Characteristics by Cognitive Status

Characteristic	Participants, No. (%)				*P* value
	Overall (N = 204)	Normal cognition (n = 52)	MCI (n = 127)	Dementia (n = 25)	
Age, mean (SD), y	70.0 (5.1)	69.9 (4.0)	69.7 (4.9)	72.1 (7.2)	.08
Sex					
Male	77 (37.7)	18 (34.6)	49 (38.6)	10 (40.0)	.86
Female	127 (62.3)	34 (65.4)	78 (61.4)	15 (60.0)	
Race					
African American	108 (52.9)	19 (36.5)	72 (56.7)	17 (68.0)	.08
White	90 (44.1)	32 (61.5)	51 (40.2)	7 (28.0)	
Ethnicity					
Hispanic or Latino	5 (2.5)	1 (1.9)	4 (3.1)	0	.62
Not Hispanic or Latino	199 (97.5)	51 (98.1)	123 (96.9)	25 (100.0)	
Years of education, mean (SD)	13.1 (2.6)	13.7 (2.8)	13.1 (2.3)	11.8 (3.2)	.01
Area Deprivation Index score, mean (SD)^a^	78.3 (19.9)	70.1 (24.5)	81.5 (15.8)	82.1 (21.7)	.006
Comorbid conditions					
Heart disease conditions^b^	47 (23.0)	10 (19.2)	27 (21.3)	10 (40.0)	.09
Cerebrovascular disease conditions^c^	20 (9.8)	1 (1.9)	13 (10.2)	6 (24.0)	.009
History of myocardial infarction	15 (7.4)	4 (7.7)	8 (6.3)	3 (12.0)	.60
Diabetes	106 (52.0)	22 (42.3)	72 (56.7)	12 (48.0)	.19
Congestive heart failure	27 (13.2)	3 (5.8)	19 (15.0)	5 (20.0)	.15
Atrial fibrillation	17 (8.3)	4 (7.7)	12 (9.4)	1 (4.0)	.65
Hypertension	190 (93.1)	45 (86.5)	121 (95.3)	24 (96.0)	.09
Dyslipidemia	42 (20.6)	8 (15.4)	30 (23.6)	4 (16.0)	.39
Cancer	47 (23.0)	11 (21.2)	31 (24.4)	5 (20.0)	.81
Chronic obstructive pulmonary disease	49 (24.0)	13 (25.0)	31 (24.4)	5 (20.0)	.88
Liver disease	14 (6.9)	4 (7.7)	8 (6.3)	2 (8.0)	.92
Chronic kidney disease	64 (31.4)	14 (26.9)	40 (31.5)	10 (40.0)	.52
Sleep apnea	61 (29.9)	12 (23.1)	45 (35.4)	4 (16.0)	.07
Insomnia	47 (23.0)	8 (15.4)	31 (24.4)	8 (32.0)	.22
Depression	75 (36.8)	22 (42.3)	45 (35.4)	8 (32.0)	.60
Anxiety disorder	50 (24.5)	13 (25.0)	31 (24.4)	6 (24.0)	.99
No. of comorbid chronic conditions, mean (SD)^d^	3.35 (1.43)	3.02 (1.60)	3.43 (1.35)	3.71 (1.35)	.11
No. of medications, mean (SD)	4.41 (2.63)	4.02 (2.69)	4.53 (2.65)	4.64 (2.48)	.45

Abbreviation: MCI, mild cognitive impairment.

^a^ Area Deprivation Index scores (range, 1-100, with higher scores denoting higher levels of neighborhood deprivation) are indexed to the national level and are the 2021 versions as obtained from the Neighborhood Atlas at UW Madison.^16^

^b^ Heart disease conditions include angina, acute myocardial infarction, or other acute or chronic ischemic heart disease.

^c^ Cerebrovascular disease conditions include transient ischemic attack (TIA), TIA-related syndromes, stroke cerebral infarction, or hemorrhagic cerebrovascular accident.

^d^ Includes heart disease, heart failure hypertension, diabetes, cancer, depression, stroke, arthritis, liver, or kidney disease.

**Table 2.** Neuropsychological and Behavioral Assessments for Participants, by Cognitive Status

Assessment^a^	Score, mean (SD)				*P* value
	Overall (N = 204)	Normal cognition (n = 52)	MCI (n = 127)	Dementia (n = 25)	
Montreal Cognitive Assessment total score	21.8 (4.2)	24.5 (2.9)	21.8 (3.2)	16.2 (5.1)	<.001
Functional Activities Questionnaire score	3.8 (6.2)	2.3 (4.7)	2.6 (4.6)	13.2 (8.1)	<.001
Clinical Dementia Rating sum of boxes	1.1 (1.4)	0.5 (0.5)	0.9 (0.8)	3.5 (2.5)	<.001
Neuropsychiatric Inventory Questionnaire score	3.8 (4.8)	2.6 (3.2)	3.6 (4.8)	7.4 (5.9)	<.001
Geriatric Depression Scale score	3 (3.0)	2.5 (2.9)	3.3 (3.2)	3.0 (1.6)	.27

Abbreviation: MCI, mild cognitive impairment.

^a^ Score ranges and definitions for all the assessments are shown in the Cognitive Assessment subsection of the Methods.

Eighty-nine of 108 African American individuals (82.4%) had undiagnosed dementia and MCI, compared with 58 of 90 White individuals (64.4%). Results from the regression models showed that African American individuals had more than twice the odds of cognitive impairment (MCI or dementia) compared with White individuals (odds ratio, 2.58; 95% CI, 1.35-5.06; *P* = .02). The association between race and cognitive impairment did not differ by age, sex, and years of education (ie, the pairwise interaction terms involving race were not statistically significant). After adjustment for age, sex, and years of education, the association remained significant (odds ratio, 2.73; 95% CI, 1.38-5.53; *P* = .02).

## Discussion

In this cross-sectional study conducted at FQHCs, we found that 62.3% of older primary care patients had unrecognized MCI, and 12.3% had unrecognized dementia. Only 41% of older adults with probable dementia both had a diagnosis and were aware of it.^18^ Unrecognized cognitive impairment is detrimental in high-risk and socially vulnerable populations, especially those with multiple other chronic conditions. Delaying diagnosis limits access to resources for treatment and care management services that enhance patients’ quality of life and increases the likelihood of poor outcomes, such as polypharmacy, falls, and abuse.^13,19^ Unrecognized cognitive impairment is also linked to poor adherence to treatment for other chronic conditions, adverse patient outcomes, and increased caregiver stress. Even though few national guidelines have found insufficient evidence for dementia screening, early diagnosis has the potential to improve patient outcomes.^20^

Several studies have investigated the reasons for high unrecognized rates of cognitive impairment in primary care and found a combination of patient-related and physician-related factors.^19^ Patient-related factors include stigma and misconceptions about cognition and dementia, other health and social need priorities, and health literacy. Physician-related factors include resource constraints related to detection, diagnostic assessment, and referral pathways; lack of training in cognitive assessment; and the need to address other pressing health conditions in patients. Our study uses detailed clinical and neuropsychological assessments to show a much higher prevalence of undiagnosed MCI and dementia in FQHC primary care sites compared with prior studies in similar populations.^21^ Our study shows persisting disparities in this area, as 82.4% of African American individuals had undiagnosed dementia compared with 64.4% of White individuals, all of whom were receiving primary care from the same FQHCs. After adjustment for confounders, African American individuals had more than double the odds of having MCI or dementia vs White individuals. The differences in diagnoses by race are likely due to both a higher prevalence of dementia in this subgroup and a lack of embedded workflows that support detection and timely diagnosis among these patients owing to patient, physician, and health care factors that need to be addressed urgently.

Our study has several strengths, including the use of both clinical and neuropsychological assessments for dementia diagnosis and a consensus review for diagnosis. The study was also done uniquely in primary care clinics (FQHCs) that served low-income people and a higher proportion of patients from underrepresented minoritized groups.

### Limitations

Our study has a few limitations, including the cross-sectional nature of the data, not having a nationally representative sample, and that the study results may not generalize to other primary care settings. Selection bias is also possible, as clinic and study participants might not reflect the true prevalence in the community. Our recruitment strategy aimed to reduce this bias by ensuring that every eligible patient at each clinic was approached. The demographics of the study population align closely with that of the overall clinic population in terms of age, sex, and race.

## Conclusions

Our study demonstrates the low rates of recognition of MCI and dementia among older adults receiving their primary care from FQHCs, particularly among patients from underrepresented minoritized groups, such as African American individuals. Incorporating implementable and scalable cognitive batteries, improving screening and diagnostic care pathways, and normalizing discussions about brain health should become a high priority in high-risk primary care populations.

## Notes

Supplement.
Data Sharing Statement
