# Error in Funding/Support Statement

**DOI:** 10.1001/jamanetworkopen.2024.52012

**Published:** 2024-11-26

In the Original Investigation titled “Prevalence of Unrecognized Cognitive Impairment in Federally Qualified Health Centers,”^1^ published October 22, 2024, the grant number in the Funding/Support statement was incorrect. This article has been corrected.^1^
