# Supplementary material for: Prevalence of Unrecognized Cognitive Impairment in Federally Qualified Health Centers
Source: JAMA Netw Open. 2024 Oct 22;7(10):e2440411. doi: 10.1001/jamanetworkopen.2024.40411 (PMC11581540; doi:10.1001/jamanetworkopen.2024.40411)
Supplement: Supplement. — Data Sharing Statement [file jamanetwopen-e2440411-s001.pdf]

Data Sharing Statement

Kulshreshtha. Prevalence of Unrecognized Cognitive Impairment in Federally Qualified Health Centers. *JAMA Netw Open*. Published October 22, 2024.
doi:10.1001/jamanetworkopen.2024.40411

Data
Data available: No

Additional Information
Explanation for why data not available: Data will be made available once the full clinical trial is completed.
